# Short-term efficacy of theta-burst stimulation for treatment resistant depression in an outpatient setting – a retrospective naturalistic study

**DOI:** 10.1192/j.eurpsy.2025.1316

**Published:** 2025-08-26

**Authors:** R. Dubickaitė, J. Montvidas

**Affiliations:** 1 Psychiatry Department, Lithuanian University of Health Sciences, Kaunas, Lithuania

## Abstract

**Introduction:**

Approximately 280 million people worldwide suffer from depression. A significant proportion of these patients (10–30%) fail to achieve full remission after receiving antidepressant treatment with at least two different antidepressant medications. These patients are treatment-resistant (Al-Harbi, 2012; Jaffe et al., 2019; Rush et al., 2006). Theta-burst stimulation (TBS) is a non-invasive brain stimulation technique where a brief magnetic field passes through the scalp to induce an electric current in the cerebral cortex in high frequencies and is used to treat treatment-resistant depression in adjunction to antidepressant treatment.

**Objectives:**

This study aimed to evaluate the short-term efficacy of TBS added to pharmacological treatment in patients with treatment-resistant depression.

**Methods:**

Patients with depression who were treatment-resistant and were treated with pharmacological treatment together with a course of 15 – 30 TBS sessions in an outpatient clinic of the Lithuanian University of Health Sciences Hospital Kaunas Clinics between 2021 and 2024 were enrolled in the study. TBS protocol stimulated in trains of 50 Hz triplets with 5 Hz frequency, a total of 20 trains in a session. The session frequency varied from once to twice daily, five days a week. The severity of depressive symptoms was evaluated with the Patient Health Questionnaire 9 (PHQ—9) before the first session of TBS and every two weeks afterward. The primary outcome measured was the mean change in the PHQ-9 score. We checked for the normality of the distribution of our variables and performed a paired sample t-test for statistical analysis of our sample.

**Results:**

40 patients were included in this study. There was a statistically significant reduction in mean PHQ-9 score following the 2-week TBS intervention (t = 6.134; 95% CI: 2.631-5.219; p < 0.001). Overall, 32 participants (80.0%) improved their PHQ-9 scores. In contrast, three participants (7.5%) showed no change in their scores, while five participants (12.5%) experienced a worsening of their symptoms. Mean pre- and post-treatment PHQ-9 scores and the mean change of the PHQ-9 score are presented in Table 1. Mean post-treatment PHQ-9 scores had no significant difference between patients whose PHQ-9 scores improved, showed no change, or worsened (p > 0.005).

**Table:** 

Outcome category	Number of patients (%)	PHQ-9 Pre-treatment, mean score (SD)	PHQ-9 post-treatment mean score (SD)	Mean PHQ-9 change (SD)	t	p
Improved	32 (80.0)	18,69 (6.44)	13,47 (6,23)	5,21 (3.39)	8.694	<0.001
Worsened	3 (7.5)	17.00 (3.24)	19.00 (3.93)	-2.0 (1.22)	-3.651	0.011
No change	5 (12.5)	15.00 (6.24)	15.00 (6.24)	0	-	-
Total	40 (100.0)	18.20 (6.10)	14.27 (6.15)	3.92 (4.04)	6.134	<0.001

**Conclusions:**

TBS is a valuable short-term intervention for patients with treatment-resistant depression in an outpatient setting.

**Disclosure of Interest:**

None Declared

